# Moving from “let’s fix them” to “actually listen”: the development of a primary care intervention for mental-physical multimorbidity

**DOI:** 10.1186/s12913-021-06307-5

**Published:** 2021-04-01

**Authors:** Kylie J. McKenzie, Susan L. Fletcher, David Pierce, Jane M. Gunn

**Affiliations:** 1 Department of General Practice, University of Melbourne, Melbourne, Australia; 2 Department of Rural Health, University of Melbourne, Ballarat, Australia; 3 Medicine, Dentistry and Health Sciences, University of Melbourne, Melbourne, Australia

**Keywords:** Multimorbidity, Intervention development, Motivational interviewing, Theory of planned behavior, Patient-centred practice, Depression

## Abstract

**Background:**

Effective person-centred interventions are needed to support people living with mental-physical multimorbidity to achieve better health and wellbeing outcomes. Depression is identified as the most common mental health condition co-occurring with a physical health condition and is the focus of this intervention development study. The aim of this study is to identify the key components needed for an effective intervention based on a clear theoretical foundation, consideration of how motivational interviewing can inform the intervention, clinical guidelines to date, and the insights of primary care nurses.

**Methods:**

A multimethod approach to intervention development involving review and integration of the theoretical principles of Theory of Planned Behavior and the patient-centred clinical skills of motivational interviewing, review of the expert consensus clinical guidelines for multimorbidity, and incorporation of a thematic analysis of group interviews with Australian nurses about their perspectives of what is needed in intervention to support people living with mental-physical multimorbidity.

**Results:**

Three mechanisms emerged from the review of theory, guidelines and practitioner perspective; the intervention needs to actively ‘engage’ patients through the development of a collaborative and empathic relationship, ‘focus’ on the patient’s priorities, and ‘empower’ people to make behaviour change.

**Conclusion:**

The outcome of the present study is a fully described primary care intervention for people living with mental-physical multimorbidity, with a particular focus on people living with depression and a physical health condition. It builds on theory, expert consensus guidelines and clinician perspective, and is to be tested in a clinical trial.

**Supplementary Information:**

The online version contains supplementary material available at 10.1186/s12913-021-06307-5.

## Background

In Australia, 1 in 4 adults are living with multimorbidity [1], or at least two chronic conditions [2]. Data from across the globe indicate that multimorbidity is a significant issue [3–6], and that people living with multimorbidity have a much higher rate of primary care utilisation [7]. An Australian study of 7620 primary care patients provided evidence that living with a higher number of chronic conditions is associated with a greater likelihood of experiencing depression [8]. There is also evidence of a bidirectional relationship for mental and physical health conditions; living with a physical health condition increases the likelihood of developing a mental health condition, and vice versa [9, 10]. People living with mental-physical multimorbidity experience poorer outcomes in terms of daily functioning, quality of life, socioeconomic deprivation, burden of illness and treatment burden compared to people living with only mental or physical conditions [4, 11].

Broad social and systemic changes to address the onset and prevalence of multimorbidity are needed [3, 12]. In countries like Australia and the UK with universal health care, primary care clinicians provide an essential frontline role. The public health implications of multimorbidity are evident in high primary care service demand [13, 14]. People living with multimorbidity have largely been excluded from the randomised controlled trials examining effective interventions for single diseases [4, 15]. As a consequence little is known about effective interventions for multimorbidity [16]. A 2016 Cochrane review by Smith and colleagues identified only sixteen trials of interventions specifically targeting people living with multimorbidity [17]. This review concluded that interventions which can be integrated into routine care and include a focus on addressing functional difficulty may be more effective [17]. Consistent with these findings, there is a call for cross-cutting interventions that promote health generally, and impact across both mental and physical health conditions [5, 18]. Recommendations also include developing interventions for multimorbidity which target positive behaviour changes to address lifestyle factors such as diet, physical activity, weight, smoking and alcohol consumption [19]. There is also a consistent call for interventions focused on patient priorities and preferences for how to improve their own health [15, 18–23]. .Evidence about public and health care interventions that address the issues of people living with mental-physical multimorbidity is limited, and effective interventions are urgently needed [24, 25].

The development of interventions for multimorbidity is a new frontier in healthcare [3]. The emerging direction for interventions to support people living with mental-physical multimorbidity is to ‘bring together’ [18] or integrate the health care needed to address both mental and physical illnesses. Primary care research recommendations about intervention for people living with depression may offer some guidance about the development of mental-physical multimorbidity interventions, especially given the high prevalence rates of comorbid depression and physical health conditions in primary care [8, 18]. Research to date supports the potential of collaborative care as a promising approach individuals with severe depression [26]. Collaborative care is defined by Gunn and colleagues as care which includes input from multiple professionals, a structured evidence-based management plan, scheduled patient follow-up, and enhanced interprofessional communication [27]. Using this definition, authors of the Cochrane review of collaborative care for depression and anxiety found collaborative care to be superior to usual care [28]. The Cochrane review authors called for researchers to provide more comprehensive descriptions of interventions including information about the ‘active ingredients’, and to include people living with long-term chronic illnesses as research participants [28]. Interview studies with people living with multimorbidity highlight the patient perspective about what is needed, including the importance of maintaining autonomy, and the significance of relationships with health professionals [11, 18, 29]. Likewise, General Practitioners identify patient-centred practice, and the time to practice that way, as key components needed in working with patients living with multimorbidity [30–32]. Recommendations for how we develop and test new interventions for multimorbidity include incorporating relevant theoretical frameworks and the perspectives of patients, clinicians, and policy makers [33, 34].

Given the potential of lifestyle behaviour change to promote well-being in both the physical and mental health domains [18], consideration of health behaviour change theory is an important factor in developing an intervention [34]. From amongst the potential candidate theories, the Theory of Planned Behavior (TPB) offers a longstanding and empirically supported foundation [35, 36] that may serve as a helpful framework for the development of interventions for multimorbidity. The TPB posits that intention to change, and actual behaviour change, are precipitated by an individual’s beliefs that: 1. The behaviour will result in the desired outcomes; 2. There is a social imperative and acceptance associated with the behaviour; and 3. The person has the ability to perform the behaviour and a sense of self-efficacy about making the behaviour change [35]. Using the TPB to guide the development of multimorbidity intervention then, would suggest that the intervention needs to support patients with evidence-based information, provide a social connection that reinforces the behaviour, as well as assisting patients to build self-efficacy [36, 37]. The outlined components of TPB also align with the recommendations for what is needed in effective interventions to support people living with multimorbidity; promoting behaviour change based on patient preference and through the development of skills and behaviours to support change in the direction of improved health outcomes [17].

Collaborative, coordinated care that does not add to treatment burden and is focused on patient priorities and cross-cutting lifestyle or health behaviour changes appears to offer the best guess as to what might be helpful for mental-physical multimorbidity intervention, based on the available evidence and qualitative research about clinician and patient perspectives [17, 25]. One approach to health care communication that may provide a skill set to address these needs and also specifically focuses on building self-efficacy for change is motivational interviewing [38, 39]. Motivational interviewing (MI) was first developed in the 1980s as an alternative to the longstanding confrontational approach taken in substance use intervention [40]. MI aims to help a person to change by strengthening the person’s own motivation for change [38]. MI is underpinned by a focus on collaboration and empathy in the helping relationship, in addition to a more technical focus that elicits and strengthens patient arguments and reasoning in support of positive behaviour change [41]. MI has been effectively applied in health care across a range of conditions, behaviour change targets, and when used by a variety of clinicians [42, 43]. This broad reach underpins the potential of MI for multimorbidity intervention [42–44].

The theoretical and clinical underpinnings of MI and the TPB appear to be congruent with expert consensus recommendations, including collaborative care approaches; that interventions for people living with mental-physical multimorbidity featuring depression need to be patient-centred, focused on health behaviour change and integrated into routine care in such a way that it does not add to treatment burden. We identified a potential role for nurses in delivering collaborative care for people living with mental-physical multimorbidity in Australian primary care settings. Nurses were selected due to the existing high demand on GP services in primary care in Australia [45, 46], the workforce of nurses in Australian primary care settings already working in chronic condition management [47, 48] and the evidence for successful delivery of MI by nurses in working with people living with chronic conditions [42]. In this paper, we describe the development of a MI-informed collaborative care intervention for people screened as at risk of experiencing severe depression in the Target-D approach to stepped care [49].. In order to develop the intervention, we drew on clinical guidelines, expert consensus recommendations, qualitative research, and theoretical considerations to address the following questions:

- What are the key components needed for an intervention for people living with mental-physical multimorbidity?
- How can motivational interviewing inform the model of care?

## Method

To develop the intervention, we:

1. Reviewed TPB as a relevant theoretical framework and its relationship with the proposed mechanisms and applied skills of motivational interviewing
2. Reviewed the intervention recommendations in expert consensus and clinical guidelines
3. Conducted group interviews with Australian nurses, and undertook analysis of the nurse perspective about interventions to support better outcomes for people living with multimorbidity

Intervention components and the overall structure of the intervention were discussed by the research team and synthesised to develop the final intervention. The intervention was then described in detail using the TIDieR template (Template for Intervention Description and Replication); a guideline designed to promote more reliable descriptions of interventions [50].

### Theory-informed development

The Theory of Planned Behavior was identified as a theory with potential to inform intervention for mental-physical multimorbidity, due to its focus on behaviour change and its broad application in behavioural outcome research [36, 51]. Motivational interviewing was identified as a candidate clinical practice to be incorporated into the intervention due to its emphasis on skills relevant to recommendations about patient-centred care and lifestyle behaviour change. The relationship between MI and TPB has also been considered by others in the development of more effective interventions [51, 52], but to our knowledge has not been reported in the literature being applied to multimorbidity intervention. The research team met and discussed the core components of TPB and MI, and their interaction, for the purposes of developing the intervention.

### Review of the clinical guidelines

Existing guidelines and Cochrane reviews for multimorbidity intervention published were identified using the bibliography of the International Research Community on Multimorbidity (http://crmcspl-blog.recherche.usherbrooke.ca/). The review included references published up until March 2016, to enable development of the intervention prior to the planned commencement of a clinical trial in 2016. Identification of potential intervention components was guided by the Foundations Framework developed by Stokes and colleagues [53]. The Foundations Framework proposes a model for designing and reporting models of care for multimorbidity. In addition to the need to define the target population and the theoretical basis of an intervention, the Foundations Framework defines three elements of intervention; the clinical focus (the clinical skills and approaches taken in the intervention), the organisation of delivery (the practical requirements needed to deliver the intervention, for example the length of the appointment), and the support for model delivery (infrastructure requirements such as necessary funding arrangements to support the delivery of the intervention). The Foundations Framework also encompasses the components of collaborative care recommended by Gunn and colleagues [27], articulated as potential examples of elements in both the clinical focus and organisation of care delivery elements of the framework. Our review focused on recommendations that fell into the categories of clinical focus, and organisation of care delivery.

### Group interviews

Two group interviews were conducted in a University-based meeting room with registered nurses who had experience working in an Australian primary care setting, which would typically include working with patients living with chronic illness and mental-physical multimorbidity. The nurses were contacted by phone and invited to participate in a group interview after expressing their interest in casual clinical nurse research assistant positions within the University’s primary care research program. Participation in the interviews was not linked to the casual employment opportunity and the researchers who conducted the interviews were not involved in the recruitment process.. Nurses completed a questionnaire to provide demographic information and background information about their qualifications and MI training experience. The questionnaire also asked nurses to rate their confidence in working with people with chronic conditions, people with mental health issues, and people with multimorbidity on a ten point scale, where are rating of ‘1’ was defined as ‘Not at all confident’ and a rating of ‘10’ was defined as ‘Very confident’ (see Electronic Supplementary Material for a copy of the questionnaire, which was developed for the study). Group interviews were conducted by KM, who is an experienced Masters level clinical psychologist, with experience conducting focus groups. KM had no prior relationship with any of the group interview participants. Participants were informed that KM was a PhD candidate investigating interventions for people living with multimorbidity.

A detailed interview topic guide was developed by KM, with feedback and discussion with JG and DP (see Electronic Supplementary Material). Broadly, the group interviews addressed the following questions:

- What do primary care nurses describe as the challenges or difficulties of working with multimorbid patients?
- What do primary care nurses describe as helpful approaches or clinical skills for working with multimorbid patients?
- What role do primary care nurses see for motivational interviewing in working with people with multimorbidity?

Analysis of the group interviews was guided by Braun and Clarke’s description of reflexive thematic analysis [54], to identify patterns of meaning to answer the questions posed. All participants in the group interviews consented to the interviews being audio-recorded. As per Clarke and Braun [55], interview recordings were listened to in full then transcribed by KM and cross-checked with the recordings. Transcripts of the interviews were uploaded to NVivo 12Plus [56]. NVivo is a software package which enables users to code text (or other data) and to link coded data points. The package was used here to facilitate review of codes and subsequent identification of themes. Initial codes were identified in relation to answering each of the broad questions; what are the challenges, what is helpful, and what is the nurse perspective of the application of MI to working with people living with multimorbidity? Themes were identified from the codes and discussed by the research team as to how well they described the nurse perspective from the interview data. In recognition of the potential bias and clinical background of the interviewer, the analysis was reviewed by DP and JG who are experienced researcher-clinicians and not MI trainers.

### Intervention development and description

**Fig. 1:**
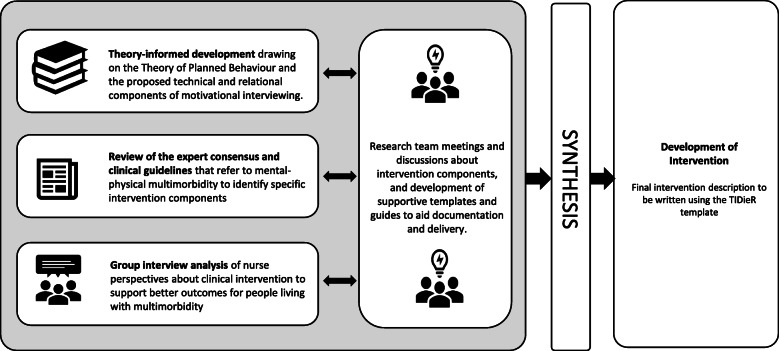
Development process for mental-physical multimorbidity intervention

The research team synthesised the available information, including the theoretical principles, nurse interviews, and guideline recommendations to determine the key components of the intervention. The TiDieR template was used to fully articulate the intervention and its components (see Fig. 1). In addition, previous observational research conducted by some of the authors of this paper was used to provide specific guidance for the skills that may be most helpful in working with patients with mental-physical multimorbidity [57].

## Results

### Theory informed development

**Fig. 2:**
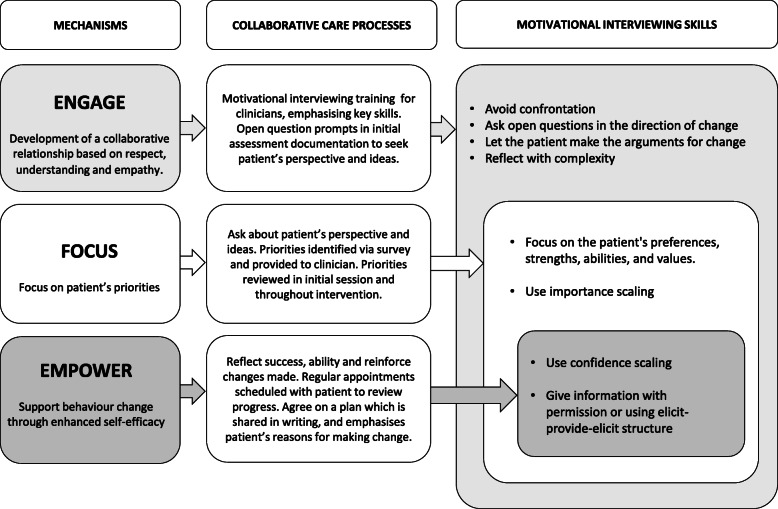
The mechanisms, skills and processes of MI-informed collaborative care

The TPB as outlined by Ajzen [35] and MI as defined my Miller and Rollnick [38] and modelled by Miller and Rose [41] were reviewed and three mechanisms were identified from the intersection of the two approaches to behaviour change to provide a theoretically-informed underpinning for the multimorbidity intervention, see Fig. 2. The three mechanisms were identified as: i) Engage: develop a collaborative relationship based on respect, understanding and empathy, ii) Focus: focus on the patient’s priorities, and iii) Empower: support behaviour change through enhanced self-efficacy. These three mechanisms highlight both the relational and technical hypotheses of motivational interviewing, that a collaborative approach paired with a deliberate focus on patient language about change may support behaviour change. The mechanisms also reflect the emphasis in the TPB on perceived behavioural control, and the role of an individual’s attitude about behaviour change and the subjective norms related to the behaviour.

### Review of the clinical guidelines

At the time of intervention development, there was a single Cochrane Review about interventions for multimorbidity [16], and three published clinical guidelines [22, 23]; one specific to mental-physical multimorbidity [18]. Using the broad structure of the Foundations Framework [53], the key clinical focus elements and organisation of delivery of care elements are summarised in Table 1. The identified clinical focus elements for multimorbidity intervention were; determining patient priority areas for intervention, the importance of a mental health focus, providing support for lifestyle behaviour change or opportunistic health promotion, and development of communication and consultation skills to support the implementation of a patient-centred intervention [18]. The elements of the intervention identified to be about the organisation of delivery were that the intervention could be integrated into routine care, delivered via longer appointments than existing GP consultation timeframes and involve scheduled review appointments, as well as enhanced communication between care providers involved in a patient’s care. The clinical focus elements identified were consistent with the theoretically derived mechanisms. The organisation of delivery elements consisted of practical considerations for the intervention, or how the intervention should best be delivered. The research team then planned the intervention with consideration of the findings, and planned strategies that would operationalize the key concepts (see Table 1).

**Table 1:** Recommendations for multimorbidity intervention and planned approach to operationalising the recommendations in the development of a new intervention

Elements of intervention	Clinical Guideline or Cochrane review offering specific support for clinical focus area	Planned approach to operationalising recommendations for intervention
*Clinical focus elements*		
Patient identified priority areas	Amer Ger Soc (2012)^a^ Muth et al. (2014)^b^ Naylor et al. (2016)^c^ Smith et al. (2012)^d^	• Provide clinicians with information about the patient’s priority areas based on survey response prior to initial appointment. • Priorities to be discussed, affirmed or altered collaboratively at the initial appointment • Planning at initial assessment and follow-up sessions to directly address patient priority areas, and this approach to be supported by documentation templates providing space for priority areas, as well as template to include helpful questions in establishing priority areas
Mental health focus	Muth et al. (2014)^b^ Naylor et al. (2016)^c^	• Intervention materials to include evidence-based behavioural targets for improving mood and well-being in people experiencing depression • Evidence-based priority areas to include: mood, anxiety, concentration, self-image, thoughts of death and concentration, health, appetite, interest, sleep, energy
Support for lifestyle behaviour change	Naylor et al. (2016)^c^ Smith et al. (2012)^d^	• Specific priority areas relevant to lifestyle behaviour change include: health, appetite, interest, sleep, energy • Intervention to include collaborative planning with patient to identify planned changes to behaviour and ongoing monitoring, review of progress and revised planning • MI communication strategies to focus on and strengthen the patient’s reasons for making changes.
Emphasis on clinical consultation skills	Amer Ger Soc (2012)^a^ Naylor et al. (2016)^c^	• All nurses participate in 2 days of training, including 1 day focused on motivational interviewing skills. • Development of support materials to reinforce MI skills. • Intervention summary plans to incorporate open questions in the templates; and intervention manual to include examples of patient-centred clinical notes to support implementation
*Organisation of delivery elements*		
Integrated into routine care	Naylor et al. (2016)^c^ Smith et al. (2013)^d^	• Program to be co-located in existing GP practices. Patients to have option of face-to-face or phone follow up sessions to promote flexibility and minimize treatment burden.
Appointments longer than GP consultation	Naylor et al. (2016)^c^	• Initial appointments planned to be up to 1-h duration, with follow up appointments up to 30 min duration. • Intervention to be delivered by primary care nurses to enable longer consultations
Scheduled review appointments	Amer Ger Soc (2012)^a^ Muth et al. (2014)^b^	• Intervention to consist of 8 sessions over approximately 12 weeks. All sessions to be scheduled collaboratively with the patient.
Enhanced communication between care providers	Muth et al. (2014)^b^ Naylor et al. (2016)^c^	• With patient consent, nurses to liaise with and share plans with GPs and other health professionals involved in the patient’s care.

^a^American Geriatrics Society Expert Panel on the Care of Older Adults with M. Guiding Principles for the Care of Older Adults with Multimorbidity: An Approach for Clinicians. J Am Geriatr Soc. 2012;60 (10):E1-E25. ^b^Muth C, van den Akker M, Blom JW, et al. The Ariadne principles: how to handle multimorbidity in primary care consultations. BMC Med 2014; 12: 223. ^c^Naylor C, Das P, Ross S, et al. Bringing together physical and mental health. King’s Fund; 2016. ^d^Smith SM, Soubhi H, Fortin M, et al. Interventions for improving outcomes in patients with multimorbidity in primary care and community settings. Cochrane Database Syst Rev. 2012;4

### Group interview analysis

#### Participants

Two group interviews were conducted with experienced primary care nurses. Three nurses attended each of the group interviews. The first interview was 33 min long and the second interview went for 42 min. All of the nurses approached agreed to participate (see Table 2 for participant characteristics). All of the nurses identified as women. The average age of the six nurses was 50.5 years (sd = 5.6, range 42–58). Three of the nurses completed their training in Australia, one in the United States, one in the United Kingdom and one in Hong Kong. On average the nurses had 28 years of practice since graduating (SD = 8.9 range: 20–40). All of the nurses were registered nurses, and three held postgraduate qualifications. All of the nurses were relatively confident working with people with chronic conditions, selecting between 7 and 10 on a 10-point confidence scale, where a rating of 1 was defined as “Not at all confident” and a rating of 10 was defined as “Very confident”. Confidence working with people with multimorbidity ranged from 6 to 8, while there was a greater spread of responses for a question about confidence working with people with mental health conditions (Range: 3–9).

**Table 2:** Participant characteristics and self-rated confidence about clinical work with people with chronic illness, mental health issues or multimorbidity

	Group 1 Mean (SD) ***n*** = 3	Group 2 Mean (SD) ***n*** = 3	Overall Mean (SD) ***n*** = 6
Age	53.00 (6.24) Range: 46–58	48.00 (5.57) Range: 42–53	50.5 (5.96) Range: 42–58
Years of practice since graduating	33.00 (8.19) Range: 24–40	23.67 (3.21) Range: 20–26	28.33 (7.55) Range: 20–40
Confidence working with people with chronic conditions	8 (1) Range: 7–9	8.67 (1.15) Range: 8–10	8.33 (1.03) Range: 7–10
Confidence working with people with mental health issues	6.67 (3.21) Range: 3–9	7.33 (1.15) Range: 6–10	7.00 (2.19) Range: 3–9
Confidence working with people with multimorbidity	7 (1) Range: 6–8	8 (0) Range: 8	7.5 (0.84) Range: 6–8

#### Analysis of group interviews

The themes identified under the broad research questions are presented below. The group interviews were engaged discussions, and the nurse participants did not express dissent with each other’s views, rather the initial individual nurse responses were endorsed and expanded upon by other group participants.

1. ***What do nurses describe as the challenges or difficulties of working with multimorbid patients?***

Four themes were identified from the group interviews about the nurses’ experiences of working with people living with multimorbidity and what they described as the challenges or difficulties of the work. The first theme identified was that behaviour change is challenging. Nurses reported that; “Change is b***dy hard work for any of us …” (Nurse 4). They identified that suggestions made by clinicians are often unrealistic, and that the expectation of a quick turnaround in changing longstanding unhelpful behaviours is likewise unrealistic. The second theme identified was that mental-physical multimorbidity is particularly challenging, Participants statements included: *“It’s not easy. There’s no simple solution for these patients and I guess when you mix the mental and the physical together it becomes slightly more complex perhaps …”* (Nurse 2). The third theme identified was that healthcare is not designed for multimorbidity, and there are systemic factors that impact on being able to work effectively with people living with multimorbidity. In particular, the nurses commented that our current system prioritises acute illness, and does not support interprofessional communication; “*… a person experiencing mental health difficulties may actually require someone to sit and listen to them for a while and to establish a rapport and trust may take longer than 6 minutes”* (Nurse 1). The fourth theme identified was that treatment burden can result from the way healthcare is structured for the management of chronic illness. Nurses described treatment burden as inherent in health care systems for chronic illnesses, such that “… you’re getting them to do stuff because you want them to be able to be billed accordingly, but really they couldn’t give a s**t about it anyway.”(Nurse 2) See Supplementary Table 1 in the Electronic supplementary material for further examples of the identified themes.

1. ***What do primary care nurses describe as helpful approaches or clinical skills for working with multimorbid patients?***

We identified six themes from the group interviews that summarise the approaches or clinical skills nurses thought would be helpful for working with people living with multimorbidity. The first three themes align with the theory-identified themes of Engage, Focus and Empower. The first identified theme was to engage with the person, to develop a collaborative relationship *( “… one of the other things I think is really important is the relationship with the patient”*; Nurse 2). The second theme was to identify and focus on patient priorities *( “… the emphasis is very often on provider priorities and we probably give too little time for patient priorities, because really from my experience unless the patient sees it as a priority it’s really not going to happen”*; Nurse 2), with nurses articulating the importance of priority areas that are identified by the patient. The third theme was to empower people, and emphasises the importance of supporting behaviour change *(“Eliciting some sort of action from them, rather than imposing your actions on people”*; Nurse 5, and *“having to temporarily suspend the nurse role of let’s fix them and let’s get a plan and do it, to actually listen …” ,* Nurse 6). Theme four highlighted the value of continuity of care; “… that’s why people like their GPs, because they go see the same person and they don’t have to have that same conversation that they had last week” (Nurse 2). A fifth theme focused on the need for helpful documentation, and nurses described the ways in which inflexible documentation processes can detract from practice; *“… sometimes training and pressure comes down from management around really strict templates and not to veer off from that, it’s quite restrictive and I found it obstructive at times.”* (Nurse 6). The sixth and final theme identified was the importance of communication between providers as an essential part of care; *“… how you’re going to share [the plan] with the other team members to make sure it’s an organised plan of support for the person that’s helpful”* (Nurse 6).

2. ***What role do Australian nurses see for motivational interviewing in working with people with multimorbidity?***

Group interview participants’ perspectives on MI suggested that the approach had face validity but was not as easy to implement in practice; *“… I seem to remember that it was all helpful and I seem to remember that it sounds really easy, and it sound very common sense, but for some reason I don’t know, I just forget to do it or something.”*(Nurse 1) The nurses spoke about the need for practice to implement the skills, and the potential utility of prompts or *“little cards and things, just as a reminder on your desk, and keeping that in mind with active listening”* (Nurse 5). Finally, the nurses in the first group raised the issue of the term MI not being meaningful in conveying what is helpful about the clinical practice of MI; *“… I mean who wants to be “interviewed”? You don’t go to the doctor to be “interviewed””(*Nurse 2). And; *“So, you know, motivational interviewing for me makes it sound like you’re imposing something on people, whereas you want them to actually change the way they do things based on their own thoughts”* (Nurse 2). The nurses interviewed considered that MI might be helpful, despite it being a term they did not relate to, and they were aware that the practice of MI takes practice.

(Verbatim responses exemplifying the identified themes are presented in the Electronic Supplementary Material).

### Relationship of the group interview analysis to the proposed theory and review of clinical guidelines

Analysis of the nurse group interviews provided support for the theory-driven concepts of engaging, focusing and empowering, and again emphasised that these theoretically derived concepts align with the clinical elements that may be required in the intervention. Consistent with the review of the guidelines, nurses also identified service delivery elements that may be important to intervention, including the importance of continuity of care, supportive documentation, and enhanced communication between providers. In addition, nurses raised the need for clinicians to be supported to develop new clinical skills, such as MI. The nurse interviews provided specific suggestions about the service delivery issues for an effective intervention, and each of these identified issues was considered and incorporated into the development of the intervention (see Table 3). Documentation and promoting enhanced communication between care providers were already incorporated into the intervention on the basis of the literature review. However, based on the input from the nurses, we reviewed draft intervention documentation following this feedback, and included examples of MI-consistent questions on the printed initial assessment documentation template as prompts, for example, “What kinds of things are important to you, which are affected by your problems in this area?” and “What ideas do you have for how you could work towards achieving your goals?”. MI-consistent prompt questions were also printed on the review session templates, for example, “How have the actions you’ve taken so far been helpful?” and “What steps might be helpful to overcome these difficulties?”. In addition, the intervention manual for nurses provided examples of how to write action plans and shared session review notes that emphasised the role of the patient at the centre of care using “You” statements, e.g. “You have walked most days this week” or “You plan to see your GP this week”. An MI Pocket Guide was developed as a quick reference tool to support nurses to use MI skills.

**Table 3:** Issues identified by nurses and corresponding intervention elements addressing issues

Issue identified by nurses	Intervention elements designed to address issues identified by nurse
Helpful documentation can support implementation	• Documentation all written with the patient at the centre of care, eg sections included “What I want to achieve” and “How I want to achieve it”. • Plans and session summaries included patient priority areas, and a brief review of progress, emphasising achievements and changes made, as well as revised plans. PHQ-9 also included in documentation and shared with patient.
Communication between providers is important	• Protocol for intervention included all plans and session summaries to be shared with patient, GP, and any other health professionals nominated by the patient. • Nurses to communicate with GPs via email after each session. • Any concerns raised with GP, and risk assessment supported by a risk assessment protocol.
Nurses need practice and prompts to develop these skills.	• MI prompts included as questions on the documentation templates. • MI Pocket Guide developed as a brief summary and prompt for MI skills. • Manual for nurses to provide a range of examples of verbal and written responses in an MI-consistent way.

### Intervention description

Integrating the information from our guideline review and nurse interviews, we developed a collaborative care intervention, designed for delivery in primary care to improve the health outcomes for people living with mental-physical multimorbidity and at risk of severe depression. The intervention was incorporated into the Target-D approach to matching depression care to individual need. As part of this approach, general practice patients complete a brief screening tool which predicts depression symptom severity and provides an opportunity to select depression treatment priorities [49]. Some of the suggested potential areas include mood, anxiety, concentration, self-image, appetite, and sleep [58]. Participants then attend up to eight sessions with a nurse trained in the intervention [49]. is intended to last approximately 60 min and involves a structured discussion around the patient’s priority areas, including setting goals and identifying actions required to make changes in those areas. Follow up sessions are planned as shorter sessions of approximately 15–30 min, and include monitoring, reinforcing of success, problem-solving and identification and referral to any additional resources or support. Following each session, the nurse provides the patient with a written summary, using a template designed to reinforce the discussion points of most importance to the participant. This written summary is also shared with the patient’s GP and other health care professionals with the patient’s permission. To engage participants and to support them to make changes to address their priority areas, nurses use MI throughout the intervention. Specifically, the skills employed are based on the description of MI by Miller and Rollnick [38], and the recommendations from an observational study of the use of MI in primary care which recommended asking more open questions, offering reflections, and avoiding confrontation in consultations [57]. To facilitate consistent use of MI, nurses are provided with a purpose-designed MI Pocket Guide resource (see Electronic Supplementary Material) and encouraged to refer to this as needed.

See Table 4 for a summary description of the intervention using the TiDieR checklist [50], or the Electronic Supplementary Material for the full TiDieR checklist.

**Table 4:** Summary description of the Collaborative Care intervention, using the Template for Intervention Description and Replication

**Target-D Collaborative Care** **WHY:** To address the need for an intervention to support people living with multimorbidity, in particular mental-physical multimorbidity. The key proposed mechanisms of the intervention, underpinned by Theory of Planned Behavior and motivational interviewing are to engage patients, focus intentionally on what is important to them, and empower them to make changes through a structured process of goal-setting, review, and individualised referral to support services and resources as appropriate. **WHO:** Patients in primary care waiting rooms complete an initial assessment and their scores indicate the likelihood of severe depression at 3 months. GPs are actively involved in the collaborative care process which is embedded in GP practices. Target-D nurses were all registered nurses. **WHAT:** ***Initial assessment:*** The initial assessment tool is an evidence-based algorithm to determine which patients are likely to experience severe depression in 3 months’ time.^a^The assessment also requires participants to select priority areas that are important to them from evidence-based areas for behaviour change to support improved mood and wellbeing, including: mood, anxiety, concentration, self-image, thoughts of death, concentration, health, appetite, interest, sleep, and energy. ***Initial collaborative care consultation:*** Target-D nurses use MI to engage participants and to collaboratively set goals and develop a written action plan focused on participant’s priority areas. ***Monitoring and review:*** Subsequent sessions continue the focus on participant priority areas, and aim to empower participants through collaborative review, providing referral or resources, and reinforcement for changes. Participants choose between face-to-face and telephone sessions. ***Follow up:*** After each session participants receive a personalised and reinforcing email and a copy of the plan. Plans are shared with the GP and health care team. Templates and plans are included in the Target-D handbook to assist Target-D nurses to write in a way that emphasises patient perspectives and supports self-efficacy. **FREQUENCY AND DURATION:** The Target-D Collaborative Care intervention provides up to 8 sessions per patient. The initial session is designed to be a longer session, proposed to last approximately 60 min, with follow up sessions planned as shorter sessions of approximately 15–30 min duration. The first four sessions are conducted weekly, and the final four sessions are conducted fortnightly. The total duration of the intervention is approximately 3 months. **TAILORING:** The intervention is driven by the priority areas identified by participants. The intervention is flexible to participant preference for face-to face or telephone sessions. Referrals to other service providers or resources are tailored to the participant’s goals and preferences.	

^a^Chondros P, Davidson S, Wolfe R, et al. Development of a prognostic model for predicting depression severity in adult primary patients with depressive symptoms using the diamond longitudinal study. Journal Affect Disord. 2018;227:854–60. doi:10.1016/j.jad.2017.11.042

## Discussion

### Summary of main findings

We developed a model for collaborative care for working with people living with mental-physical multimorbidity, by integrating theoretical understandings, key clinical review evidence, and target clinician insights. We have identified key mechanisms, processes and skills for delivering collaborative care for people living with mental-physical multimorbidity. The intervention is designed to enhance both engagement and action in patients with mental-physical multimorbidity. The intervention incorporates key ideas about supporting behaviour change from TPB, as well as the clinical skills and approach of MI, and organisation delivery elements that have been recommended in the expert consensus and clinical guidelines. In addition, the perspectives of nurses about the challenges of the work and what is needed to work effectively with this patient group provided crucial insights into the necessary components of the intervention from those who will deliver it on the ground. Our analysis found the nurses’ perspectives to be consistent with the research and clinical guidelines. The nurse perspectives further supported the broad ideas about the mechanisms of collaborative care; to engage, focus on patient priorities and empower and support behaviour change. The review of the guidelines and the group interview analysis also reinforced the need for structuring the intervention with the collaborative care nurses having access to intervention support materials and templates designed to act as a supportive guide for the implementation of the intervention. This guiding approach to intervention development is consistent with MI [38], and also enables the patient-centred flexibility that is sometimes difficult to implement with manualized interventions [59]. From our initial framework we were able to develop a comprehensive approach to collaborative care, and to develop materials for the collaborative care nurses to support implementation of the intervention.

### Relating the findings to existing literature

This intervention is aimed at supporting behaviour change by addressing the priority areas selected by intervention participants. Implementation and evaluation of this intervention may add to the existing literature about coordinated or collaborative care, by examining the impact of specific behaviour change support. The TrueBlue randomised cluster trial of a nurse-led model of collaborative care in Australia provides initial evidence for the role of collaborative care interventions in addressing depression in primary care [60], although the trial was affected by clinic withdrawal resulting in 28% (*n* = 36) patients withdrawing from the intervention arm. Other coordinated care interventions such as the six-monthly review appointments of the 3D trial in the UK [61] do not include ongoing behavioural support for the plans developed as part of the program. Evaluating the proposed MI-informed collaborative care approach may provide helpful information about more targeted behaviour support for patients with mental-physical multimorbidity, identification of the intervention elements that work, and those that do not, and information about how to optimise collaborative care for delivery in routine care.

### Future directions

The MI-informed collaborative care model described in this paper has been trialed with primary care patients identified as being likely to experience severe and chronic depressive symptoms in the Target-D randomised controlled trial [49]. Evaluation of the collaborative care intervention as part of the Target-D trial may provide insights about the implementation of collaborative care in real world settings, as well as assist in answering the questions about intervention mechanisms and contexts to contribute to an improved understanding of what works for people living with mental-physical multimorbidity [62].

### Strengths and limitations

One of the strengths of the development of the Collaborative care intervention is the consideration of theoretical foundations. While there is a clear need for the development of new interventions for mental-physical multimorbidity [63], there has also been a call for clinicians and researchers to develop interventions with consideration of the theoretical constructs that may underpin them [34]. In considering the potential for MI in primary care, Keeley and colleagues specifically ask about how theories of behaviour change might be used to contribute to more effective implementation of MI [44]. Evaluation of the proposed mechanisms of the collaborative care intervention may provide helpful information about the mechanisms of effective interventions for mental-physical multimorbidity. A further strength of this study is the use of the TIDieR framework to describe and define the intervention.

One of the limitations of this study is that the review of guidelines was conducted in 2016, and the availability of research and guidelines at the time of intervention development was limited. However, this is also a reflection of the limited research in the area generally, and in 2018 Baker and colleagues discuss the paucity of intervention research in multimorbidity [63]. This is further realized in the limited number of studies included (*n* = 18) in the Smith and colleagues Cochrane Systematic review of interventions for improving outcomes in patients with multimorbidity in primary care and community settings [17]. The intervention described has a particular focus on depression as the most prevalent condition associated with mental-physical multimorbidity, and this may represent a limitation to its potential application to a broader range of mental health conditions. Additionally, the group interviews conducted as part of this study were with a small group of nurses based in a large metropolitan area who were recruited due to their pre-existing interest in primary care research. Although the interviews provided rich and relevant material for the development of the intervention, the recruitment process may have limited the scope of nurse perspectives. The intervention development was informed by the qualitative research about patient and clinician experience of multimorbidity but may certainly have benefited from interviewing GPs and people living with multimorbidity directly and involving patients and primary care clinicians in the development of the intervention.

## Conclusion

There is a call from multimorbidity researchers for intervention studies to better articulate and evaluate the components of interventions reported, how they work, and for whom they work [63]. This paper presents the development of primary care intervention for people living with mental-physical multimorbidity, with a particular focus on people living with depression and a physical health condition. The key components of the intervention are outlined in detail, providing a framework to support evaluation.

## Supplementary Information

**Additional file 1: Supplementary material.** Questionnaire for nurses.

**Additional file 2: Supplementary material.** Topic guide for group interviews with nurses.

**Additional file 3: Supplementary material.** Verbatim responses and identified themes from group interviews with nurses. **Supplementary Table 1**: Themes in the group interview data related to challenges of multimorbidity intervention and quotes exemplifying the themes. **Supplementary Table 2**: Nurse perspectives about what is helpful in working with people with multimorbidity. **Supplementary Table 3**: Nurse perspectives about motivational interviewing and its application to multimorbidity.

**Additional file 4: Supplementary material.** TIDieR: Template for Intervention Description and Replication.

**Additional file 5: Supplementary material.** The Motivational Interviewing (MI) Pocket Guide resource to support skill development in MI-informed collaborative care; Side 1.

## Data Availability

Three additional tables providing more detailed interview data are available in the supplementary information files. Due to ethical concerns, the interview transcripts cannot be made publicly available. All other data generated or analysed during this study are included in this published article and its supplementary information files.

## Abbreviations

MI: motivational interviewing
TIDieR: Template for Intervention Description and Replication
TPB: Theory of Planned Behavior
