# Supplementary material for: Moving from “let’s fix them” to “actually listen”: the development of a primary care intervention for mental-physical multimorbidity
Source: BMC Health Serv Res. 2021 Apr 1;21:301. doi: 10.1186/s12913-021-06307-5 (PMC8017734; doi:10.1186/s12913-021-06307-5)
Supplement: Supplementary file 1 — Additional file 1: Supplementary material. Questionnaire for nurses. [file 12913_2021_6307_MOESM1_ESM.docx]

**SUPPLEMENTARY MATERIAL: Questionnaire for nurses**

| Please complete this survey about you and your practice. All information you give us is strictly confidential and all findings from this survey will be presented in an anonymous form. |
| --- |

**1**  Are you male or female? □Male □Female

**2**  What is your age? ______________ years

**3**  In what year did you become a registered nurse/clinician? ______________ year

**4**  Country of graduation?

□ Australia □ New Zealand □ United Kingdom

□Other (please specify) ________________________________

**5**  What are your qualifications?

_______________________________________________________

**6** Please circle:

**a)** How confident are you working with people with chronic conditions?

1 2 3 4 5 6 7 8 9 10

| Not at all confident |  | Very  confident |
| --- | --- | --- |

**b)** How confident are you working with people with mental health issues?

1 2 3 4 5 6 7 8 9 10

| Not at all confident |  | Very  confident |
| --- | --- | --- |

**c)** How confident are you working with people with multimorbidity?

1 2 3 4 5 6 7 8 9 10

| Not at all confident |  | Very  confident |
| --- | --- | --- |

**7** What training/experience have you had in motivational interviewing?

□ No previous training

□ I’ve attended an information session or read about it

□ I’ve participated in a ½ day workshop

□ I’ve participated in a 1 day workshop

□ I’ve participated in 2 or more days of workshop training

□ I’ve undertaken advanced training or supervision

**Thank you**
