# Supplementary figures and images for: Moving from “let’s fix them” to “actually listen”: the development of a primary care intervention for mental-physical multimorbidity
Source: BMC Health Serv Res. 2021 Apr 1;21:301. doi: 10.1186/s12913-021-06307-5 (PMC8017734; doi:10.1186/s12913-021-06307-5)

**SUPPLEMENTARY MATERIAL: Topic guide for group interviews with nurses**


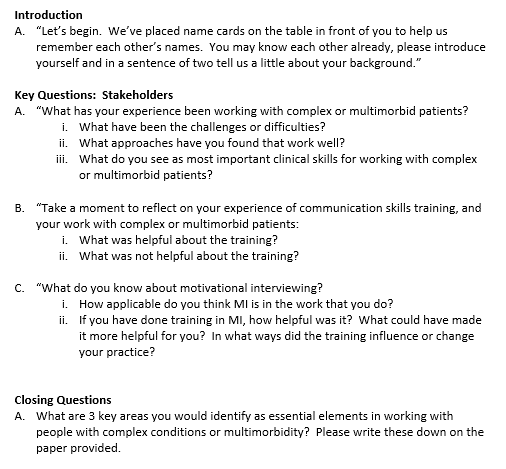

Supplement: Supplementary file 2 — Additional file 2: Supplementary material. Topic guide for group interviews with nurses. [file 12913_2021_6307_MOESM2_ESM.docx]
