# Supplementary material for: Moving from “let’s fix them” to “actually listen”: the development of a primary care intervention for mental-physical multimorbidity
Source: BMC Health Serv Res. 2021 Apr 1;21:301. doi: 10.1186/s12913-021-06307-5 (PMC8017734; doi:10.1186/s12913-021-06307-5)
Supplement: Supplementary file 3 — Additional file 3: Supplementary material. Verbatim responses and identified themes from group interviews with nurses. Supplementary Table 1: Themes in the group interview data related to challenges of multimorbidity intervention and quotes exemplifying the themes. Supplementary Table 2: Nurse perspectives about what is helpful in working with people with multimorbidity. Supplementary Table 3: Nurse perspectives about motivational interviewing and its application to multimorbidity. [file 12913_2021_6307_MOESM3_ESM.docx]

**SUPPLEMENTARY MATERIAL:** Verbatim responses and identified themes from group interviews with nurses

*Supplementary Table 1: Themes in the group interview data related to challenges of multimorbidity intervention and quotes exemplifying the themes*

| Behaviour change is challenging. | Mental-physical multimorbidity is particularly challenging | Healthcare is not designed for multimorbidity, and it impacts on being able to work most effectively with multimorbid patients. | Treatment burden can result from the way healthcare is structured for chronic illness |
| --- | --- | --- | --- |
| N5: Any suggestions that you might make, they’ve always got something that “no, but” or “yes, but” and I find that sometimes is really frustrating that just having a simple conversation with someone, that it’s really difficult to elicit some positivity in that half an hour to an hour..  N4: Change is bloody hard work for any of us – you know, goals are often unrealistic, so you’re doomed to fail from the start. I do it myself, I’ll go on a diet and I’ll lose 10 kilos. You know it’s do as I say not as I do.  N4: Absolutely, people are years in getting to where they are and it will be years in reversing that, and sometimes that’s just too hard to even contemplate.  N5: – they don’t expect to have any changes. Often they just think “just carry on regardless” and they usually come back with other issues, either environmental, financial, relationships.  N5: And that gets bigger and bigger – so it’s steeple chasing. | N2: It’s not easy. There’s no simple solution for these patients and I guess when you mix the mental and the physical together it becomes slightly more complex perhaps and when you mix things in like lack of access to certain providers, it becomes more complicated…  N1: and you’re seeing someone for 8 minutes, 11 minutes, and 6 minutes and that particularly perhaps a person experiencing mental health difficulties may actually require someone to sit and listen to them for a while and to establish a rapport and trust may take longer than 6 minutes. | N1: Yeah, once again it goes back to the funding model, I guess. And you can have long appointments, but in the meantime the waiting room might be having someone come in that’s fainted, a broken arm…you know, people have run in and out of the room, the doctor’s been interrupted, or the nurse has been interrupted three times to do an ECG, give an immunisation. It’s that actually uninterrupted focused time so that that person can actually talk to you.  N3: … I think sometimes I lack really a full understanding of how the patient is, having complex needs or comorbidities, …we do not have understanding of what … the clients are doing with other discipline.  N1: I’d say the disconnection and the lack of coordination of care is a challenge to the patient’s outcomes…they have multiple complex chronic illnesses and the psychologist doesn’t know what the GP is doing, who doesn’t know what the physio’s doing. I think that’s part of the challenge I see. | N2: “Do I need a review of my cycle of care? I don’t care.” But the practice needs it to be sustainable. So mixing those things or trying to work with them is difficult.  N2: and then you mix in things like funding requirements and care plans – that adds another layer of complexity. Because you’re getting them to do stuff because you want them to be able to be billed accordingly, but really they couldn’t give a s… about it anyway.  N1: The funding model can actually be detrimental to the outcome you’re actually seeking, which is hopefully to help the patient to achieve the best health that they can achieve or support them in whatever state that they choose to be. |

N: nurse, number indicates the nurse who made the comments

Supplementary Table 2: Nurse perspectives about what is helpful in working with people with multimorbidity

| Engage: Developing a collaborative relationship based on respect, understanding and empathy | Focus: Identify and focus on patient’s priorities | Empower: Support behaviour change through enhanced self-efficacy | Continuity of care | Helpful documentation can support implementation | Communication between providers is important |
| --- | --- | --- | --- | --- | --- |
| N1: I think time is really important. … you’re seeing someone for 8 minutes, 11 minutes, and 6 minutes … perhaps a person experiencing mental health difficulties may actually require someone to sit and listen to them for a while and to establish a rapport and trust may take longer than 6 minutes.  N2: … one of the other things I think is really important is the relationship with the patient.  N1: We’re talking about partnerships, patients, consumers or clients or whatever term you prefer. Communication is a two-way thing; it’s not just imparting the preferred line of treatment.  N3: …you do deliver a different message, whether you focus more on an uneven relationship with the client or encouraging her to take up the role of being the carer for herself, there’s a lot of things to do with your attitude.  N1: It’s that actually uninterrupted focused time so that that person can actually talk to you. | N1: But how often as health care providers do we ask patients what it is they want to get out of it and where they see themselves being. I’m not actually sure. I’d like to think it happens all the time. You know if we’re talking about patient-centred care and patients setting their own goals and us working with them to help them to achieve those goals.  N2: Well I think that unfortunately the emphasis is very often on provider priorities and we probably give too little time for patient priorities – because really from my experience unless the patient sees it as a priority it’s really not going to happen.  .  N6: Prioritising what’s actually meaningful and most relevant to the person and being creative around how my agenda and maybe their agenda can actually marry. | N5: Eliciting some sort of action from them, rather than imposing my actions on people.  N4: Correct, it’s fun and great for me to say “Well, take this medicine four times a day”. But if they don’t have a refrigerator, or they can’t store the antibiotics, well that’s going to come to grief. So it’s like actually having a conversation about how is this going to be managed. So, that back and forth – the two-way thing.  N6: Yes, and there’s something about having to temporarily suspend I guess the nurse role of let’s fix them and let’s get a plan and do it, to actually listen, and that takes time which sometimes we’re poor on. And sometimes more than one go as well, you know, of trying to get to the heart of it, of what is it about that that’s impacting on them that’s then going to help them find the motivation to actually care enough to make whatever choices that they need to consider.  N3: We should know what kind of resources that’s available to the client, how to coordinate that kind of care. | N2: Yeah, and that’s why people like their GPs, because they go and see the same person and they don’t have to have that same conversation that they had last week. There’s a certain level of understanding about what point they might be at on their continuum of care.  N3: That also puts to my mind the contact point. One contact point will coordinate the care and also know the level of what her condition is. So, I think it is more clear with one point of contact. | N6: Sometimes trainings and pressure that come down through management around efficiency and around really strict templates, and not to veer off from that, it’s quite restrictive and I found it obstructive at times to actually getting to what you’re trying to accomplish.  N4: … you’ve got a template and do your assessment, but it’s still what do you want? What can you get out of today? Why are you here? | N6: I would say the things that have already been named, so the active listening, you know patient-centred care, having a collaborative approach, how you’re going to share it with the other team members to make sure it’s an organised plan of support for the person that’s helpful.  N2: Well, I think when it’s done well, it is truly a collaboration and it’s respectful, it’s meaningful, it’s relevant, it’s recognised as meaningful. |

N: nurse, number indicates the nurse who made the comments

Supplementary Table 3: Nurse perspectives about motivational interviewing and its application to multimorbidity

| The application of MI to working with complex patients has face validity | Nurses need practice and prompts to develop MI skills. | The term “motivational interviewing” is not helpful in conveying what it is. |
| --- | --- | --- |
| N1: I can’t actually remember, but I have done a bit of motivational interviewing training and I seem to remember that it was all helpful, and I seem to remember that it sounds really easy but, and it sounds very common sense, but for some reason I don’t know, I just forget to do it, or something. | What might help do you think to implement it for people?  N2: Um, I don’t know really, but I guess practice, and being able to practise it on an ongoing basis is really important, but I don’t know why that’s so hard.  N1: I think we’re talking about embedding it, aren’t we? So that practising it once doesn’t mean you’re a master in the art, and you probably need support to reinforce those skills.  N2: You need an acronym that says “Ask, Listen, Reflect” or something like that.  N5: Having little cards and things, just as a reminder on your desk, and keeping that in mind with active listening. | N2: Yeah, I don’t know why that is, I mean the mere term of it “motivational interviewing” is a bit of a funny one anyway, because, you know, it is about communication – why we call it “interviewing”- I mean who wants to be “interviewed”? You don’t go to the doctor to be “interviewed”.  N1: Is there another term that you could use that would be inspirational – inspirational discussions, you know you’re trying to inspire people to make changes based on their own reflective thoughts.  N2: So, you know, motivational interviewing for me makes it sound like you’re imposing something on people, whereas you want them to actually change the way they do things based on their own thoughts. |

N: nurse, number indicates the nurse who made the comments
