# Supplementary material for: Moving from “let’s fix them” to “actually listen”: the development of a primary care intervention for mental-physical multimorbidity
Source: BMC Health Serv Res. 2021 Apr 1;21:301. doi: 10.1186/s12913-021-06307-5 (PMC8017734; doi:10.1186/s12913-021-06307-5)
Supplement: Supplementary file 4 — Additional file 4: Supplementary material. TIDieR: Template for Intervention Description and Replication. [file 12913_2021_6307_MOESM4_ESM.docx]

**SUPPLEMENTARY MATERIAL: TIDieR: Template for Intervention Description and Replication**

**NAME OF INTERVENTION**

Target-D Collaborative Care

**WHY**

The rationale for this intervention is an urgent need for the development of interventions that address the complexity of multimorbidity, and in particular mental-physical multimorbidity.

The key theories underpinning this intervention are the Theory of Planned Behavior, and the proposed theoretical underpinnings of motivational interviewing as an approach to supporting behaviour change (see Figure 1). TPB posits that behaviour change is influenced by the attitude a person holds about the performance of a behaviour and its likely outcome, the normative beliefs about the behaviour and the social pressure or support to perform the behaviour and the individual’s own perceived self-efficacy about being able to perform the behaviour; their belief about their ability to do so. This collaborative care intervention aims to address this by supporting multimorbid patients to make lifestyle changes that promote improved health outcomes through an intervention that applies the principles of motivational interviewing; engaging patients, focusing intentionally on what is important to them, and empowering them to make changes through a structured process of goal-setting, review and individualised referral to support services and resources as appropriate.

**WHAT**

**Training**

The nurses participate in a two-day training program; 1 day focused on the evidence underpinning the program, and the Collaborative care resources and processes, which are outlined in the handbook for the nurses, and 1 day of motivational interviewing skills training.

Consistent with MI; training focused on specific guidance to avoid the righting reflex, and take an exploring, eliciting stance in collaborative care. Key skills emphasised were:

- Avoiding the righting reflex and confrontation
- Asking open questions in the direction of change
- Use of complex reflection
- Providing advice with permission or using elicit-provide-elicit framework
- Use of importance/confidence rulers

Nurses receive training materials and an MI Pocket Guide to reinforce these skills.

**Intervention**

See pictorial representation of intervention in Figure 2.

*Participants:*

Patients referred for care complete the *diamond* Clinical Prediction Tool (CPT) and their scores indicated the likelihood of severe depression at 3 months. The CPT is an evidence-based algorithm to determine which patients are likely to be experiencing severe depression in three months’ time(1,2). More detailed information about the CPT and inclusion criteria is reported in Gunn et al (2017)(1). The target population for the intervention is people presenting in primary care with clinically significant mental health needs, including those with physical comorbidities.

*Use of Clinical Prediction Tool*

A brief self-assessment based on the CPT is completed on a digital tablet in the GP waiting room. The assessment result is provided to the Target-D nurse with the priority focus areas identified by the participant. The evidence-based areas for behaviour change to support improved mood and wellbeing include: mood, anxiety, concentration, self-image, thoughts of death and concentration, health, appetite, interest, sleep, energy. For patients randomised to the intervention arm of the severe depression group, the priority areas identified by patients were populated into the initial assessment materials provided to Collaborative care nurses.

*Initial Assessment*

The initial assessment is a consultation between the Target-D nurse and patient. The aim of the initial assessment is development of an action plan to improve mood, quality of life and self-efficacy, focused on the individual’s priority areas. The action plan template for the initial session provides open questions within the document to promote engagement and support the use of a MI approach. Questions such as *What does a typical day involve for you?* are included to provide patients with the opportunity to talk about their lives and the impact of their health conditions, with Target-D nurses responding to patients with active listening skills. Priority areas are formally written into the initial assessment plan, and questions such as *What ideas do you have about how you could work towards achieving your goal?* form part of the written plan, again with the aim of engaging patients collaboratively.

*Monitoring and Review*

Subsequent sessions are designed to provide patients with a continued focus on their priority areas, as well as conversations to empower them to make changes, by providing monitoring, review, feedback, resources and reinforcement for changes made and next steps. Questions on the monitoring and review form include *How have you been going with the actions you planned to take? How have the actions you’ve taken so far been helpful?*  The PHQ-9 is administered at each review session and shared with patients and their health professionals.

*Follow up*

After each session with a Target-D nurse the intervention participants receive a personalised and reinforcing email with copies of the initial assessment and plan after the first session, and a copy of the Progress Review and Plan for each follow-up appointment attended. Example emails and plans are included in the Target-D handbook to assist Target-D nurses to write the plans in a way that emphasises patient perspectives and supports self-efficacy. Target-D nurses communicate with GPs and other providers about the collaborative care intervention and plans also.

Materials:

- Target-D handbook
- Initial session assessment and action plan template
- Session by session Progress Review and Plan template
- MI pocket guide
- Training Materials
- Interview documentation
- Shared plans

**WHO PROVIDES**

GPs are actively involved in the Collaborative care process which is embedded in GP practices. Target-D nurses were all registered nurses.

**HOW**

The intervention is provided individually. Patients who consent to participate in the intervention are provided with a choice about whether the sessions are conducted face to face or over the phone.

**WHERE**

Target-D nurses deliver the intervention from the GP clinics where patients attend their GPs for face-to-face consultations. Target-D nurses are able to conduct telephone consultations in the clinics, or from another location. Target-D nurses are provided with both a laptop computer and a mobile phone.

**WHEN and HOW MUCH**

The collaborative care intervention provides up to 8 sessions per patient. The initial session is designed to be a longer session, proposed to last approximately 60 mins, with follow up sessions planned as shorter sessions of approximately 15-30 minutes duration. The first four sessions are conducted weekly, and the final four sessions are fortnightly. The total duration of the intervention is approximately 3 months.

**TAILORING**

The intervention is driven by the priority areas identified by patients. The intervention is flexible to patient preference for face -to face or telephone sessions. In addition, patients are able to flexibly schedule appointments at suitable intervals to meet their needs. Referrals to other service providers or resources are tailored to the patient’s goals and preferences. Patients are also able to withdraw or discontinue collaborative care at any point of the intervention.

**MODIFICATIONS**

The intervention was not modified during the course of the study.

**HOW WELL**

Planned intervention fidelity assessments include motivational interviewing fidelity assessment using the OnePass(3). Target-D nurses were asked to audio record interventions to facilitate fidelity checks, with patient consent. Intervention fidelity is also planned to assess the match between patient priority areas and subsequent shared patient plans.


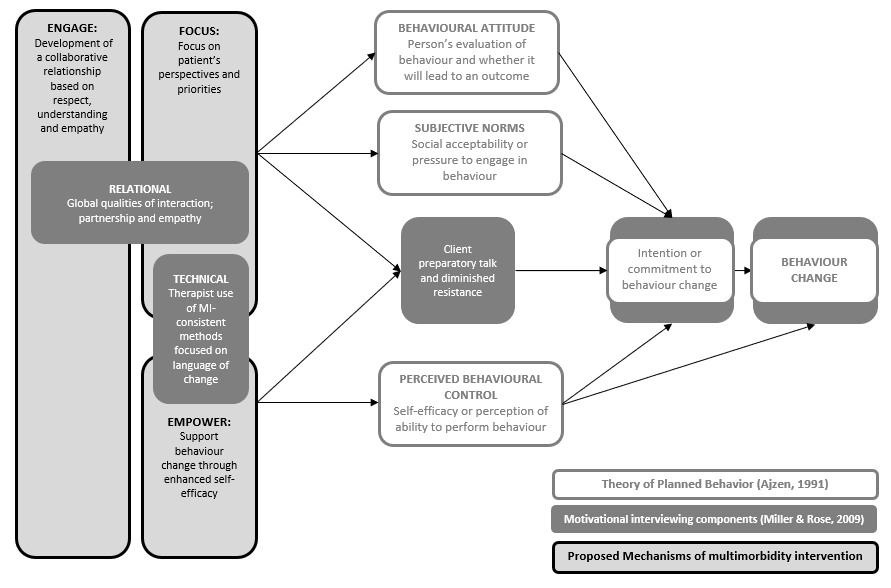


**Figure 1:** Identified mechanisms for multimorbidity intervention based on MI and TPB


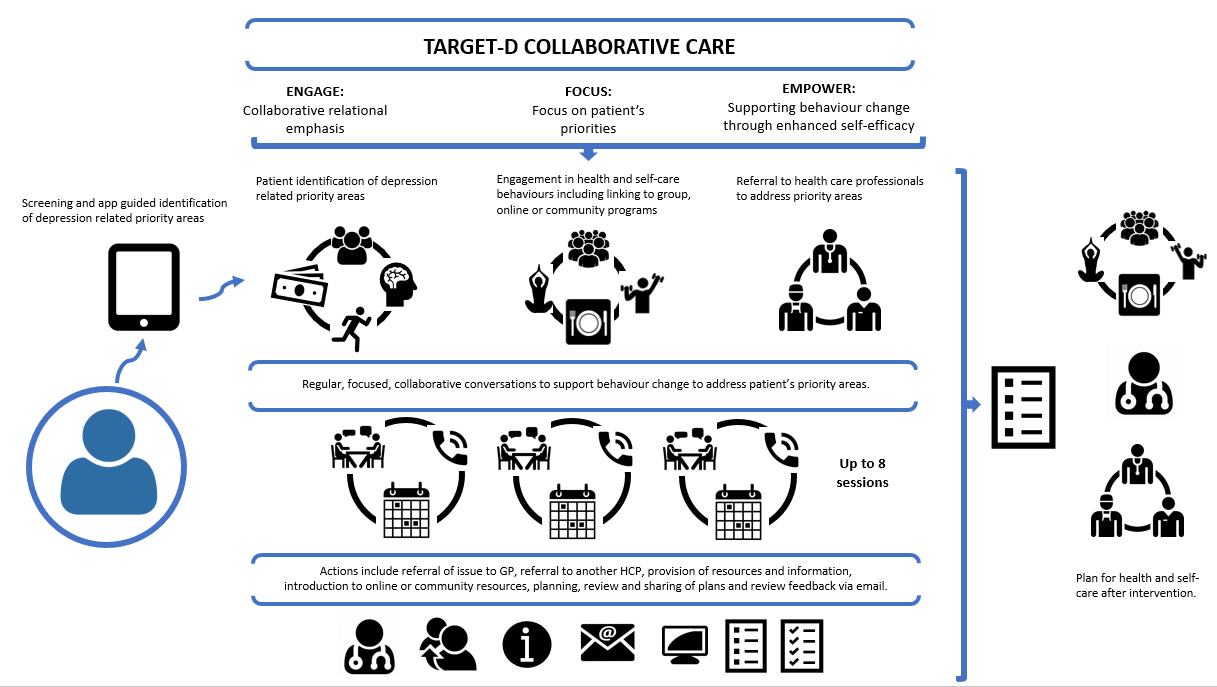


**Figure 2:** Pictorial representation of the Collaborative Care intervention

**REFERENCES**

1. Gunn J, Wachtler C, Fletcher S, et al. Target-D: a stratified individually randomized controlled trial of the diamond clinical prediction tool to triage and target treatment for depressive symptoms in general practice: study protocol for a randomized controlled trial. Trials. 2017;18(1):342.

2. Chondros P, Davidson S, Wolfe R, et al. Development of a prognostic model for predicting depression severity in adult primary patients with depressive symptoms using the diamond longitudinal study. Journal Affect Disord. 2018;227:854-60. doi:10.1016/j.jad.2017.11.042

3. McMaster F, Resnicow K. Assessment: Validation of the one pass measure for motivational interviewing competence. Patient Educ Couns. 2015;98:499-505. doi:10.1016/j.pec.2014.12.014
