# Supplementary material for: Moving from “let’s fix them” to “actually listen”: the development of a primary care intervention for mental-physical multimorbidity
Source: BMC Health Serv Res. 2021 Apr 1;21:301. doi: 10.1186/s12913-021-06307-5 (PMC8017734; doi:10.1186/s12913-021-06307-5)
Supplement: Supplementary file 5 — Additional file 5: Supplementary material. The Motivational Interviewing (MI) Pocket Guide resource to support skill development in MI-informed collaborative care; Side 1. [file 12913_2021_6307_MOESM5_ESM.docx]

**SUPPLEMENTARY MATERIAL:** *The Motivational Interviewing (MI) Pocket Guide resource to support skill development in MI-informed collaborative care; Side 1*


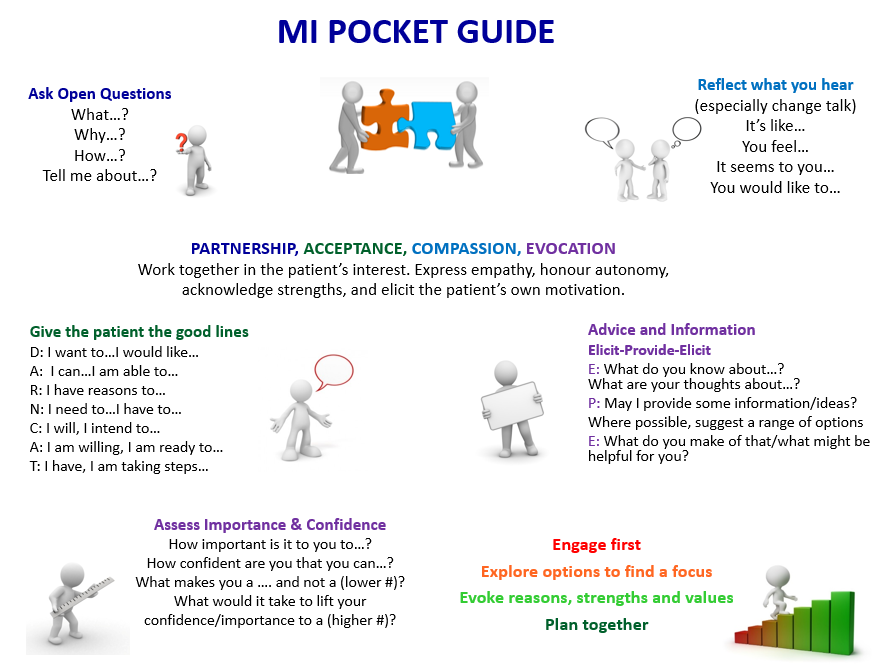


**SUPPLEMENTARY MATERIAL:** *MI-informed ‘Elicit-Provide-Elicit’ structure to support collaborative care goal-setting process (printed on the back of the MI Pocket Guide)*

**GOAL SETTING**

| **WHAT I WANT TO ACHIEVE** | |
| --- | --- |
| *Elicit:* | What kinds of things are important to you, which are affected by your problems in this area?  What would you like to be doing, that you’re finding difficult at the moment because of these issues? |
| *Provide:* | Would you like me to provide some suggestions that other people have found useful? |
| *Elicit:* | For each of your priority areas, what do you think you would like to focus on? |
| **HOW I WANT TO ACHIEVE IT** | |
| *Elicit:* | What ideas do you have for how you could work towards achieving your goals?  What do you know about the options that are available to help? |
| *Provide:* | Would you like me to provide some suggestions about actions that other people have found useful? |
| *Elicit:* | What action(s) would you be most comfortable and confident with taking? |
| **THINGS I NEED TO GET STARTED**  Are there any special referrals or resources you need to take action towards meeting your goals in your priority areas? | |
